# Abnormal nutritional status determined by the controlling nutritional status score is associated with lymph node metastasis in female patients with breast cancer, but Naples prognostic score not

**DOI:** 10.3389/fonc.2026.1771942

**Published:** 2026-03-23

**Authors:** Weihua Wen, Litao Jin, Yuyang Yang, Jianjuan Li

**Affiliations:** Department of Breast Surgery, Meizhou People’s Hospital, Meizhou Academy of Medical Sciences, Meizhou, China

**Keywords:** breast cancer, control of nutritional status score, lymph node metastasis, Naples prognostic score, relationship

## Abstract

**Background:**

Nutritional status and inflammatory status play crucial role in the occurrence and development of cancer. This study aims to explore the relationship between abnormal nutritional status determined by the Control Nutritional Status (CONUT) score, Naples Prognostic Score (NPS) and the lymph node metastasis (LNM) in female patients with breast cancer.

**Methods:**

A retrospective analysis was conducted on the clinical data (demographic information, medical history, and pathological features) of 627 female patients with breast cancer. The patients were divided into the LNM group and the non-LNM group based on the status of LNM. The CONUT score and NPS score of all patients were calculated, and the relationship between LNM and the CONUT score and NPS score was analyzed.

**Results:**

There were 280 (44.7%) patients with LNM and 347 (55.3%) without LNM. The proportions of NPS >1 (56.4% vs. 27.7%, *p* < 0.001) and CONUT abnormal (34.6% vs. 10.7%, *p* < 0.001) in patients with LNM were higher than those in patients without LNM. There was no statistically significant difference in age (*p* = 0.678), BMI (*p* = 0.399), hypertension (*p* = 0.137), family history of cancer (*p* = 0.435), and laterality of cancer (*p* = 0.872) between the two groups. Logistic regression analysis showed that abnormal nutritional status determined by the CONUT score (odds ratio (OR): 2.147, 95% confidence interval (CI): 1.304-3.534, *p* = 0.003) is independently associated with LNM, whereas NPS is not associated with LNM (*p* = 0.084).

**Conclusions:**

Abnormal nutritional status determined by the CONUT score is associated with LNM in female patients with breast cancer, but NPS not.

## 1 Introduction

Breast cancer is a malignant tumor that originates from the epithelial tissue of the breast (1). Its pathological characteristics mainly manifest as abnormal proliferation of breast cells under the influence of various carcinogenic factors, losing normal regulation and forming local masses, with the potential to invade surrounding tissues and metastasize to distant sites (2). From a global perspective, breast cancer has become a major public health issue seriously threatening the health of women, and the burden of the disease is extremely heavy. According to the latest cancer statistics, the incidence rate and mortality rate of breast cancer rank second and fourth respectively (3). In China, with the development of the economy and society as well as the acceleration of the aging process of the population, the incidence of breast cancer has been increasing year by year, and the age at which it occurs is gradually getting younger (4, 5).

Lymph node metastasis (LNM) of breast cancer refers to the process where breast cancer cells break away from the primary tumor site, spread through the lymphatic circulation system to regional lymph nodes and form secondary tumors (6, 7). It is one of the common metastasis methods during the progression of breast cancer. LNM of breast cancer significantly increases the complexity and intensity of treatment for patients (8, 9), as well as the risk of complications (10). Moreover, in terms of prognosis, LNM is one of the important factors affecting the survival rate of breast cancer patients. In terms of mechanism, LNM of breast cancer is a complex biological process involving multiple steps and multiple factors, it encompasses interactions among tumor cell characteristics (11), tumor microenvironment (12), and lymphatic circulation system (13), and so on.

In recent years, numerous studies have shown that the nutritional status of patients and the inflammatory state within their bodies play a crucial role in the occurrence and development of cancer (14, 15). The Control of Nutritional Status (CONUT) score is a scoring system that comprehensively assesses serum albumin, cholesterol, and lymphocyte counts to evaluate the nutritional and immune status of patients (16, 17). Some studies have shown that the CONUT score was closely related to the prognosis of various cancers (18–21). However, the association between it and LNM in breast cancer patients still requires further in-depth exploration. The Naples Prognostic Score (NPS) is a scoring system that comprehensively reflects the nutritional and inflammatory immune status of patients by evaluating serum albumin levels, total cholesterol levels, the ratio of neutrophils to lymphocytes, and the ratio of lymphocytes to monocytes (22, 23). In some tumors, NPS has been proven to have certain prognostic predictive value (22, 24, 25). However, the relationship between NPS and LNM in breast cancer is still unclear. The purpose of this study was to evaluate the relationship of CONUT, NPS and LNM in breast cancer patients. This study should provide additional valuable reference data for diagnosis and treatment options for breast cancer patients.

## 2 Materials and methods

### 2.1 Participants

This study was a retrospective cohort study. The research subjects were selected from breast cancer patients who received treatment in our hospital from July 2017 to November 2024. Inclusion criteria (1): patients diagnosed with primary breast cancer through pathological tissue examination (2); had not received anti-tumor treatments such as radiotherapy, chemotherapy, or targeted therapy before surgery; and (3) complete clinical and follow-up data, including demographic information, pathological features, laboratory test results. Exclusion criteria (1): patients complicated with other malignant tumors (2); with severe liver or kidney dysfunction, autoimmune diseases, and infectious diseases that affect nutritional status and inflammatory indicators; and (3) patients with incomplete clinical data. A total of 627 female patients meeting the criteria were finally included. This study was supported by the Ethics Committee of the Meizhou People’s Hospital.

### 2.2 Data collection

Clinical data of patients were collected through the hospital’s electronic medical record system, including (1): demographic information: age, gender (all in this study were female), and body mass index (BMI) (2); medical history: history of hypertension, diabetes, and family history of cancer (3); pathological features: tumor location (left breast, right breast, and bilateral), TNM stage, molecular subtypes (luminal A, luminal B, human epidermal growth factor receptor 2 (HER2) enriched (HER2+), and triple negative breast cancer (TNBC)) (26), LNM status, and distant metastasis status (4); laboratory test indicators: preoperative serum albumin, lymphocyte count, total cholesterol, neutrophil count, and monocyte count.

In this study, patients were divided into two groups according to age: ≤55 years old and >55 years old. BMI was divided into three grades: underweight (<18.5 kg/m^2^), normal weight (18.5-23.9 kg/m^2^), and overweight (≥24.0 kg/m^2^) (27, 28). The diagnostic criteria of diabetes mellitus as follows: random serum glucose ≥11.1mmol/L; or fasting blood glucose (FBG) ≥7mmol/L; or blood glucose level at the 2-hour oral glucose tolerance test ≥11.1mmol/L (29). The diagnostic criteria of hypertension as follows: mean SBP >140 mmHg and/or mean DBP >90 mmHg (30). The clinical staging was conducted using the breast cancer tumor-node-metastasis (TNM) staging system developed by the American Joint Committee on Cancer (AJCC) (8th edition) (31). LNM of breast cancer was mainly evaluated by assessing the involvement of regional lymph nodes. The examined regional lymph nodes included axillary lymph nodes (levels I–III), internal mammary lymph nodes, and infraclavicular lymph nodes.

### 2.3 Data processing and statistical analysis

#### 2.3.1 CONUT score

The preoperative serum albumin concentration, peripheral lymphocyte count, and total cholesterol level of the patients were scored as follows:

1. the serum albumin: ①≥35g/L: 0 point, ②30-34.9g/L: 2 points, ③25-29.9g/L: 4 points, and ④<25g/L: 6 points;
2. peripheral lymphocyte count: ①≥1.6 × 10^9^ count/L: 0 point, ②1.2-1.59 × 10^9^ count/L: 1 point, ③0.8-1.19 × 10^9^ count/L: 2 points, and ④<0.8 × 10^9^ count/L: 3 points;
3. total cholesterol: ①>180mg/dL: 0 point, ②140-180mg/dL: 1 point, ③≥100 and <140mg/dL: 2 points, and ④<100mg/dL: 3 points.

CONUT score is the sum of the scores of three indicators: total lymphocyte count in peripheral blood, serum albumin and total cholesterol level. Degree of malnutrition based on the CONUT score (1):normal: 0–1 point (2), light: 2–4 points (3), moderate: 5–8 points, and (4) severe: 9–12 points (32). This study referred to some previous studies and defined the CONUT 2–12 points as the high-risk group (the CONUT abnormal group) (33–35). The definition of abnormal CONUT status (CONUT 2–12 points) was determined according to the most widely accepted and clinically validated cutoff in previous large-scale studies. This threshold has been extensively used in oncology nutrition research, represents a clinically meaningful deterioration in nutritional condition, which is closely associated with systemic inflammation, impaired immune function, and adverse oncologic outcomes.

#### 2.3.2 NPS score

In this study, NPS was defined as: ①serum albumin (≥40 g/L: 0 point, <40 g/L: 1 point); ②total cholesterol (>180mg/dL: 0 point, ≤180mg/dL: 1 point); ③neutrophil to lymphocyte ratio (NLR) (≤2.96: 0 point, >2.96: 1 point); ④lymphocyte to monocyte ratio (LMR) (>4.44: point, ≤4.44: 1 point) (36). NPS score is the sum of the scores of albumin, total cholesterol, NLR, and LMR. NPS were categorized into 3 groups: group 0 (score of 0), group 1 (score 1 or 2), and group 2 (score of 3 or 4) (37). In this study, the cutoff of NPS >1 as the high-risk group, consistent with the majority of clinical studies focusing on prognostic or metastatic stratification in solid tumors. This cutoff effectively identifies patients with a combined disadvantage in nutritional and inflammatory markers, and has been validated in prognostic stratification and survival analyses in some cancers (38–40).

### 2.4 Statistical analysis

Data analysis was performed using SPSS statistical software version 26.0 (IBM Inc., USA). Categorical variables are expressed as the number of cases (%), and compared using the χ^2^ test. Logistic regression analysis was used to analysis the relationship of CONUT, NPS and LNM risk. In the multivariate analysis, the associations between NPS, CONUT and LNM were analyzed after adjusting for age, BMI, hypertension, family history of cancer, laterality of breast cancer, and molecular subtypes as confounding factors. *p* < 0.05 was considered statistically significant.

## 3 Results

### 3.1 Clinicopathological features of patients

There were 395 (63.0%) patients aged ≤55 years old and 232 (37.0%) cases aged >55 years old. There were 287 (45.8%), 23 (3.7%), and 317 (50.6%) patients with normal weight, underweight, and overweight based on BMI, respectively. There were 129 (20.6%), and 44 (7.0%) patients had hypertension, and family history of cancer, respectively. The proportions of luminal A, luminal B, HER2+, and TNBC was 11.8% (74/627), 33.5% (210/627), 12.8% (80/627), and 12.1% (76/627), respectively. The number of patients with NPS of 0, 1, 2, 3 and 4 was 143 (22.8%), 230 (36.7%), 172 (27.4%), 64 (10.2%), and 18 (2.9%), respectively. There were 493 (78.6%), 127 (20.3%), and 7 (1.1%) patients with normal, mild, and moderate malnutrition according to CONUT score, and there was no patient with severe malnutrition (**Table 1**).

**Table 1:** The clinical features of all patients.

Clinicopathological features	Total (n=627)
Age (years)	
≤55, n (%)	395 (63.0%)
>55, n (%)	232 (37.0%)
BMI (kg/m^2^)	
Underweight, n (%)	23 (3.7%)
Normal weight, n (%)	287 (45.8%)
Overweight, n (%)	317 (50.6%)
Hypertension	
No, n (%)	498 (79.4%)
Yes, n (%)	129 (20.6%)
Family history of cancer	
No, n (%)	583 (93.0%)
Yes, n (%)	44 (7.0%)
Laterality of breast cancer	
Left, n (%)	319 (50.9%)
Right, n (%)	308 (49.1%)
TNM stage	
I-II, n (%)	455 (72.6%)
III-IV, n (%)	172 (27.4%)
Lymph node metastasis	
No, n (%)	347 (55.3%)
Yes, n (%)	280 (44.7%)
Distant metastasis	
No, n (%)	580 (92.5%)
Yes, n (%)	47 (7.5%)
Molecular subtypes	
Luminal A, n (%)	74 (11.8%)
Luminal B, n (%)	210 (33.5%)
HER2+, n (%)	80 (12.8%)
TNBC, n (%)	76 (12.1%)
Unknown, n (%)	187 (29.8%)
NPS	
0	143 (22.8%)
1	230 (36.7%)
2	172 (27.4%)
3	64 (10.2%)
4	18 (2.9%)
CONUT grades	
Normal, n (%)	493 (78.6%)
Mild, n (%)	127 (20.3%)
Moderate, n (%)	7 (1.1%)
Severe, n (%)	0 (0)

BMI, body mass index; TNM, tumor-node-metastasis; TNBC, triple-negative breast cancer; HER2, human epidermal growth factor receptor 2; NPS, naples prognostic score; CONUT, controlling nutritional status; IQR, interquartile range.

### 3.2 Comparison of clinical features among patients with and without LNM

There were 280 (44.7%) patients with LNM and 347 (55.3%) without LNM. The proportion of luminal B molecular subtype (45.4% vs. 23.9%, *p* = 0.005) in patients with LNM was higher than that in patients without LNM. The proportions of NPS >1 (56.4% vs. 27.7%, *p* < 0.001) and CONUT abnormal (34.6% vs. 10.7%, *p* < 0.001) in patients with LNM were higher than those in patients without LNM. There was no statistically significant difference in age (*p* = 0.678), BMI (*p* = 0.399), hypertension (*p* = 0.137), family history of cancer (*p* = 0.435), and laterality of cancer (*p* = 0.872) between the two groups (**Table 2**).

**Table 2:** Comparison of clinical features among patients with or without lymph node metastasis.

Clinicopathological features	Patients without lymph node metastasis (n=347)	Patients with lymph node metastasis (n=280)	*p* (χ^2^)
Age (years)			
≤55, n (%)	216 (62.2%)	179 (63.9%)	0.678 (χ^2^ = 0.188)
>55, n (%)	131 (37.8%)	101 (36.1%)	
BMI (kg/m^2^)			
Underweight, n (%)	12 (3.5%)	11 (3.9%)	0.399 (χ^2^ = 1.895)
Normal weight, n (%)	151 (43.5%)	136 (48.6%)	
Overweight, n (%)	184 (53.0%)	133 (47.5%)	
Hypertension			
No, n (%)	268 (77.2%)	230 (82.1%)	0.137 (χ^2^ = 2.286)
Yes, n (%)	79 (22.8%)	50 (17.9%)	
Family history of cancer			
No, n (%)	320 (92.2%)	263 (93.9%)	0.435 (χ^2^ = 0.694)
Yes, n (%)	27 (7.8%)	17 (6.1%)	
Laterality of breast cancer			
Left, n (%)	178 (51.3%)	141 (50.4%)	0.872 (χ^2^ = 0.055)
Right, n (%)	169 (48.7%)	139 (49.6%)	
TNM stage			
I-II, n (%)	341 (98.3%)	114 (40.7%)	<0.001 (χ^2^ = 257.873)
III-IV, n (%)	6 (1.7%)	166 (59.3%)	
Molecular subtypes			
Luminal A, n (%)	45 (13.0%)	29 (10.4%)	0.005 (χ^2^ = 12.638)
Luminal B, n (%)	83 (23.9%)	127 (45.4%)	
HER2+, n (%)	40 (11.5%)	40 (14.3%)	
TNBC, n (%)	42 (12.1%)	34 (12.1%)	
NPS			
≤1	251 (72.3%)	122 (43.6%)	<0.001 (χ^2^ = 53.196)
>1	96 (27.7%)	158 (56.4%)	
CONUT grades			
Normal, n (%)	310 (89.3%)	183 (65.4%)	<0.001 (χ^2^ = 53.722)
Mild, n (%)	34 (9.8%)	93 (33.2%)	
Moderate, n (%)	3 (0.9%)	4 (1.4%)	

BMI, body mass index; TNM, tumor-node-metastasis; TNBC, triple-negative breast cancer; HER2, human epidermal growth factor receptor 2; NPS, naples prognostic score; CONUT, controlling nutritional status; IQR, interquartile range.

### 3.3 Comparison of clinical features among patients with and without LNM in patients with NPS ≤1 and >1, respectively

In patients with NPS ≤ 1 (n=373), there were 251 (67.3%) cases without LNM and 122 (32.7%) with LNM. There was no statistically significant difference in age (*p* = 1.000), BMI (*p* = 0.469), hypertension (*p* = 0.303), family history of cancer (*p* = 1.000), laterality of cancer (*p* = 1.000), and molecular subtypes (*p* = 0.279) between the two groups (**Table 3**).

**Table 3:** Comparison of clinical features among patients with and without LNM in patients with NPS ≤ 1 and >1, respectively.

Clinicopathological features	NPS ≤ 1 (n=373)			NPS>1 (n=254)		
	Non-lymph node metastasis (n=251)	Lymph node metastasis (n=122)	*p* (χ^2^)	Non-lymph node metastasis (n=96)	Lymph node metastasis (n=158)	*p* (χ^2^)
Age (years)						
≤55, n (%)	144 (57.4%)	70 (57.4%)	1.000 (χ^2^ = 0.001)	72 (75.0%)	109 (69.0%)	0.321 (χ^2^ = 1.054)
>55, n (%)	107 (42.6%)	52 (42.6%)		24 (25.0%)	49 (31.0%)	
BMI (kg/m^2^)						
Underweight, n (%)	8 (3.2%)	7 (5.7%)	0.469 (χ^2^ = 1.516)	4 (4.2%)	4 (2.5%)	0.526 (χ^2^ = 1.369)
Normal weight, n (%)	107 (42.6%)	53 (43.4%)		44 (45.8%)	83 (52.5%)	
Overweight, n (%)	136 (54.2%)	62 (50.8%)		48 (50.0%)	71 (44.9%)	
Hypertension						
No, n (%)	187 (74.5%)	97 (79.5%)	0.303 (χ^2^ = 1.133)	81 (84.4%)	133 (84.2%)	1.000 (χ^2^ = 0.002)
Yes, n (%)	64 (25.5%)	25 (20.5%)		15 (15.6%)	25 (15.8%)	
Family history of cancer						
No, n (%)	232 (92.4%)	113 (92.6%)	1.000 (χ^2^ = 0.004)	88 (91.7%)	150 (94.9%)	0.425 (χ^2^ = 1.082)
Yes, n (%)	19 (7.6%)	9 (7.4%)		8 (8.3%)	8 (5.1%)	
Laterality of breast cancer						
Left, n (%)	129 (51.4%)	62 (50.8%)	1.000 (χ^2^ = 0.011)	49 (51.0%)	79 (50.0%)	0.898 (χ^2^ = 0.026)
Right, n (%)	122 (48.6%)	60 (49.2%)		47 (49.0%)	79 (50.0%)	
TNM stage						
I-II, n (%)	247 (98.4%)	57 (46.7%)	<0.001 (χ^2^ = 145.462)	94 (97.9%)	57 (36.1%)	<0.001 (χ^2^ = 94.732)
III-IV, n (%)	4 (1.6%)	65 (53.3%)		2 (2.1%)	101 (63.9%)	
Molecular subtypes						
Luminal A, n (%)	26 (10.4%)	18 (14.8%)	0.279 (χ^2^ = 3.841)	19 (19.8%)	11 (7.0%)	0.003 (χ^2^ = 13.644)
Luminal B, n (%)	61 (24.3%)	67 (54.9%)		22 (22.9%)	60 (38.0%)	
HER2+, n (%)	29 (11.6%)	23 (18.9%)		11 (11.5%)	17 (10.8%)	
TNBC, n (%)	24 (9.6%)	14 (11.5%)		18 (18.8%)	20 (12.7%)	

BMI, body mass index; TNM, tumor-node-metastasis; TNBC, triple-negative breast cancer; HER2, human epidermal growth factor receptor 2; NPS, naples prognostic score.

Among patients with NPS >1 (n=254), there were 96 (37.8%) cases without LNM and 158 (62.2%) with LNM. The distribution of molecular subtypes was statistically significant difference in patients with and without LNM (*p=*0.003). There was no statistically significant difference in age (*p* = 0.321), BMI (*p* = 0.526), hypertension (*p* = 1.000), family history of cancer (*p* = 0.425), and laterality of cancer (*p* = 0.898) between the two groups (**Table 3**).

### 3.4 Comparison of clinical features among patients with and without LNM in patients with CONUT normal and CONUT abnormal, respectively

In patients with CONUT normal (n=493), there were 310 (62.9%) cases without LNM and 183 (37.1%) with LNM. The distribution of molecular subtypes was statistically significant difference between the two groups (*p=*0.015). There was no statistically significant difference in age (*p* = 0.924), BMI (*p* = 0.447), hypertension (*p* = 0.116), family history of cancer (*p* = 0.587), and laterality of cancer (*p* = 0.577) between the two groups (**Table 4**).

**Table 4:** Comparison of clinical features among patients with and without LNM in patients with CONUT normal and CONUT abnormal, respectively.

Clinicopathological features	CONUT normal (n=493)			CONUT abnormal (n=134)		
	Non-lymph node metastasis (n=310)	Lymph node metastasis (n=183)	*p* (χ^2^)	Non-lymph node metastasis (n=37)	Lymph node metastasis (n=97)	*p* (χ^2^)
Age (years)						
≤55, n (%)	186 (60.0%)	111 (60.7%)	0.924 (χ^2^ = 0.021)	30 (81.1%)	68 (70.1%)	0.276 (χ^2^ = 1.643)
>55, n (%)	124 (40.0%)	72 (39.3%)		7 (18.9%)	29 (29.9%)	
BMI (kg/m^2^)						
Underweight, n (%)	10 (3.2%)	9 (4.9%)	0.447 (χ^2^ = 1.610)	2 (5.4%)	2 (2.1%)	0.713 (χ^2^ = 1.053)
Normal weight, n (%)	131 (42.3%)	83 (45.4%)		20 (54.1%)	53 (54.6%)	
Overweight, n (%)	169 (54.5%)	91 (49.7%)		15 (40.5%)	42 (43.3%)	
Hypertension						
No, n (%)	234 (75.5%)	150 (82.0%)	0.116 (χ^2^ = 2.809)	34 (91.9%)	80 (82.5%)	0.192 (χ^2^ = 1.871)
Yes, n (%)	76 (24.5%)	33 (18.0%)		3 (8.1%)	17 (17.5%)	
Family history of cancer						
No, n (%)	287 (92.6%)	172 (94.0%)	0.587 (χ^2^ = 0.355)	33 (89.2%)	91 (93.8%)	0.462 (χ^2^ = 0.830)
Yes, n (%)	23 (7.4%)	11 (6.0%)		4 (10.8%)	6 (6.2%)	
Laterality of breast cancer						
Left, n (%)	156 (50.3%)	87 (47.5%)	0.577 (χ^2^ = 0.356)	22 (59.50%)	54 (55.7%)	0.703 (χ^2^ = 0.157)
Right, n (%)	154 (49.7%)	96 (52.5%)		15 (40.5%)	43 (44.3%)	
TNM stage						
I-II, n (%)	305 (98.4%)	80 (43.7%)	<0.001 (χ^2^ = 201.045)	36 (97.3%)	34 (35.1%)	<0.001 (χ^2^ = 41.593)
III-IV, n (%)	5 (1.6%)	103 (56.3%)		1 (2.7%)	63 (64.9%)	
Molecular subtypes						
Luminal A, n (%)	36 (11.6%)	22 (12.0%)	0.015 (χ^2^ = 10.475)	9 (24.3%)	7 (7.2%)	0.149 (χ^2^ = 5.410)
Luminal B, n (%)	69 (22.3%)	89 (48.6%)		14 (37.8%)	38 (39.2%)	
HER2+, n (%)	35 (11.3%)	25 (13.7%)		5 (13.5%)	15 (15.5%)	
TNBC, n (%)	36 (11.6%)	21 (11.5%)		6 (16.2%)	13 (13.4%)	

BMI, body mass index; TNM, tumor-node-metastasis; TNBC, triple-negative breast cancer; HER2, human epidermal growth factor receptor 2; CONUT, controlling nutritional status.

In patients with CONUT abnormal (n=134), there was no statistically significant difference in age (*p* = 0.276), BMI (*p* = 0.713), hypertension (*p* = 0.192), family history of cancer (*p* = 0.462), laterality of cancer (*p* = 0.703), and molecular subtypes (*p* = 0.149) between the two groups (**Table 4**).

### 3.5 Logistic regression analysis of the relationship between NPS, CONUT and LNM in breast cancer patients

The results of univariate analysis showed that NPS >1 (odds ratio (OR): 3.386, 95% confidence interval (CI): 2.426-4.726, *p* < 0.001), and abnormal nutritional status determined by the CONUT score (OR: 4.441, 95% CI: 2.917-6.762, *p* < 0.001) were significantly associated with LNM in breast cancer patients. Multivariate regression logistic analysis showed that abnormal nutritional status determined by the CONUT score (OR: 2.147, 95% CI: 1.304-3.534, *p* = 0.003) remained independently associated with LNM, whereas NPS was not associated with LNM (*p* = 0.084) (**Table 5**).

**Table 5:** Logistic regression analysis of the relationship between NPS, CONUT and LNM in breast cancer patients.

Variables	Univariate		Multivariate	
	OR (95% CI)	*p* values	OR (95% CI)	*p* values
Age (>55 vs. ≤55, years old)	0.930 (0.671-1.290)	0.665	1.137 (0.744-1.740)	0.553
BMI (kg/m^2^)				
Normal weight	1.000 (reference)	–	1.000 (reference)	–
Underweight	1.018 (0.435-2.382)	0.968	0.743 (0.276-1.997)	0.555
Overweight	0.803 (0.582-1.107)	0.180	1.019 (0.679-1.528)	0.928
Hypertension (yes vs. no)	0.737 (0.497-1.095)	0.131	1.215 (0.700-2.106)	0.489
Family history of cancer (yes vs. no)	0.766 (0.409-1.436)	0.406	0.784 (0.384-1.601)	0.504
Laterality of breast cancer (right vs. left)	1.038 (0.758-1.423)	0.815	1.212 (0.819-1.793)	0.337
Molecular subtypes (non-luminal vs. luminal)	0.740 (0.501-1.095)	0.133	0.725 (0.482-1.090)	0.122
NPS (>1 vs. ≤1)	3.386 (2.426-4.726)	<0.001	1.454 (0.951-2.223)	0.084
CONUT (abnormal vs. normal)	4.441 (2.917-6.762)	<0.001	2.147 (1.304-3.534)	0.003

BMI, body mass index; TNM, tumor-node-metastasis; NPS, naples prognostic score; CONUT, controlling nutritional status.

To further ensure the robustness of our results, we also performed sensitivity analysis using alternative cutoffs (such as NPS >0 and >2, CONUT ≥1 and ≥3). The main conclusion remained unchanged: abnormal CONUT status was significantly associated with LNM, whereas NPS was not, supporting the stability of our findings (**Table 6**). Owing to the limited number of patients with NPS >3 and CONUT ≥4, further analysis based on this cutoff was not feasible.

**Table 6:** Logistic regression analysis of the association of different cut-off values of NPS and CONUT with LNM in breast cancer patients.

Variables	Crude β/OR (95% CI)	*p* values	Adjusted β/OR (95% CI)	*p* values
NPS				
NPS (>0 vs. 0)	2.533 (1.687-3.802)	<0.001	1.401 (0.816-2.406)	0.222
NPS (>1 vs. ≤1)	3.386 (2.426-4.726)	<0.001	1.454 (0.951-2.223)	0.084
NPS (>2 vs. ≤2)	2.410 (1.490-3.897)	<0.001	1.277 (0.745-2.191)	0.374
CONUT				
CONUT (≥1 vs. <1)	2.789 (2.011-3.870)	<0.001	1.851 (1.250-2.741)	0.002
CONUT (≥2 vs. <2)	4.441 (2.917-6.762)	<0.001	2.147 (1.304-3.534)	0.003
CONUT (≥3 vs. <3)	4.348 (2.098-9.012)	<0.001	2.238 (1.025-4.888)	0.043

NPS, naples prognostic score; CONUT, controlling nutritional status; OR, odds ratio; CI, confidence interval.

Adjust for: age, BMI, hypertension, family history of cancer, laterality of breast cancer, and molecular subtypes.

## 4 Discussion

In breast cancer, patients with LNM often face a higher risk of recurrence and a lower survival rate (41). Therefore, identifying indicators that can effectively predict the risk of LNM in breast cancer patients is of great clinical significance for formulating individualized treatment plans and improving patient prognosis. This study found that the CONUT score was significantly associated with LNM in breast cancer patients, while the NPS did not show a similar association. This result provides a new perspective for the risk assessment of LNM in breast cancer and also prompts in-depth thinking about the differences in the roles of the two nutrition-inflammation scores in tumor progression.

The CONUT score, as a comprehensive indicator integrating serum albumin, total cholesterol, and lymphocyte count, can fully reflect the body’s nutritional reserves and immune status (42). In this study, its significant relationship with LNM is consistent with the conclusions of previous multiple studies on the impact of nutrition-immune status on progression in some cancers (43, 44). However, some studies have shown that there was no relationship between CONUT and LNM of biliary tract cancer (45), gynecological cancer (46), pancreatic cancer (47), and medullary thyroid cancer (48). Existing research has confirmed that malnutrition can enhance the invasive and metastatic abilities of tumor cells by reducing the immune surveillance function, altering the tumor microenvironment (such as promoting hypoxia and abnormal angiogenesis), and so on (49, 50). Specifically, a decrease in serum albumin levels may weaken the vascular endothelial barrier function, creating conditions for tumor cells to penetrate the lymph vessel wall (51). Total cholesterol, as an important component of the cell membrane, has an abnormal level that may affect the fluidity and invasiveness of tumor cells (52, 53). A decrease in lymphocyte count directly weakens the body’s ability to clear tumor cells, making it easier for tumor cells to metastasize through the lymph circulation (54, 55).

In the field of breast cancer, previous studies have shown that preoperative nutritional risk is related to tumor stage and the degree of lymph node involvement (56, 57). The CONUT score, as a more objective quantitative indicator, further indicates its association with LNM, suggesting that the nutritional-immune status may be an important regulatory factor for the invasive biological behavior of breast cancer (58, 59). The clinical significance of this finding lies in the fact that the CONUT score can serve as a simple and feasible prognostic assessment tool, helping clinicians identify patients with a high risk of LNM, and subsequently formulate individualized treatment and nutritional support plans to improve patient prognosis. Some studies have consistently shown that high CONUT score is associated with poor survival period in breast cancer patients (58–62). Li et al. found that high CONUT was associated with lymph node involvement in patients with breast cancer (61). The research conducted by Zhang et al. indicates that the CONUT score is an independent risk factor for postoperative lung infection in breast cancer patients (63).

The results of this study indicate that the NPS has no significant relationship with LNM in breast cancer patients. NPS is composed of inflammatory indicators and serum albumin and cholesterol indicators. Its core logic is to assess the prognosis of the disease through the “inflammation-nutrition” systemic state (22, 23). However, LNM in breast cancer is a highly dependent biological process on the local microenvironment, mainly involving the adhesion of tumor cells to the endothelial cells of lymph sinuses, the formation of a local immunosuppressive microenvironment, and local events such as lymphangiogenesis (64). Studies have shown that the formation of LNM depends on the interaction between the chemotactic factors secreted by tumor cells and the local lymphocytes, rather than changes in the concentration of systemic inflammatory factors (65, 66). Moreover, serum albumin, as a marker of nutritional status, its decreased level may indirectly reflect the overall consumption of the body, and has a relatively low direct relationship with the direct migration of tumor cells through the lymphatic channels (67). Therefore, the “inflammation-nutrition” status evaluated by NPS, which represents the systemic condition, lacks a close pathological physiological connection with the local biological behavior of lymph node metastases. This is the core reason for the lack of significant association between the two. Previous studies have suggested that NPS is related to the prognosis of breast cancer patients. Xiu et al. found that NPS is a biomarker related to the prognosis of breast cancer patients who have received neoadjuvant chemotherapy (68). High NPS score is associated with poor prognosis of breast cancer (69, 70). In addition, the NPS was significantly associated with LNM in univariate analysis, but not in multivariate analysis. This discrepancy may be attributed to the confounding effects of other clinicopathological factors, such as tumor stage, tumor size, histological grade, and tumor microenvironment-related factors, which may overlap with the prognostic and predictive value of NPS. NPS reflect the systemic inflammatory and nutritional status of patients, which could interact with tumor biology and disease progression. After adjusting for major clinicopathological characteristics, the independent predictive value of NPS for LNM was attenuated, suggesting that NPS may serve as a complementary indicator rather than an independent predictive factor for LNM in breast cancer. These findings highlight the importance of comprehensive evaluation of inflammatory-nutritional scores combined with traditional pathological factors in clinical practice.

The differences between CONUT and NPS in the assessment of LNM in breast cancer stem from the fact that they evaluate the body state from different dimensions: CONUT focuses more on the “nutrition-immunity” dual state, and the lymphocyte counts it includes are directly related to the anti-tumor immune function. While NPS leans more toward the “inflammation-nutrition” combined state, with higher weights given to NLR and LMR. This dimensional difference may lead to the differentiation of the predictive efficacy of the two for different clinical outcomes. For LNM that relies on immune escape and nutritional support, CONUT has higher sensitivity. While NPS may have a greater advantage in evaluating the long-term prognosis related to systemic inflammation (such as distant metastasis and survival time). Moreover, sample characteristics may also affect the results. The patients included in this study were mainly with early-stage breast cancer, and the influence of the local tumor microenvironment may be more significant, while the systemic inflammatory response has not been fully activated, which may further weaken the association of NPS. Future studies can expand the sample size and include patients of different stages to verify this hypothesis.

The study has some limitations that are worth noting. Firstly, all patients were enrolled from a single Chinese center, and the study population consisted exclusively of females. Regional differences in genetic background, lifestyle, dietary habits, and ethnic characteristics may lead to disparities in tumor biology and prognosis. In addition, variations in healthcare systems, screening strategies, and clinical management protocols across different regions and countries may further restrict the broader generalizability of the present results. Therefore, our findings should be applied with appropriate caution, mainly to populations with similar demographic and clinical characteristics. Secondly, this is a single-center retrospective study with a limited sample size, which may introduce selection bias. And the results are observational and exploratory, without evidence for causality. Therefore, the preliminary predictive value of the CONUT score requires further validation in prospective, multi-center, large-cohort studies before clinical application can be recommended. The results need to be verified by multi-center prospective studies. Thirdly, due to the retrospective nature of the study design, some critical clinicopathological variables, including tumor size, histological grade, and lymphovascular invasion, were either unavailable or incompletely documented in the database. Consequently, these well−recognized predictors of lymph node metastasis could not be incorporated into the multivariable regression models. This may have led to residual confounding and restricted the comprehensiveness of the adjusted analyses. Finally, the stratified analysis considering factors such as breast cancer molecular typing and tumor location was not included, which may affect the accuracy of the results.

Future research can be conducted as follows: First, increase the sample size and conduct multi-center studies to verify the predictive value of the CONUT score for LNM in breast cancer. Second, combine single-cell sequencing, immunohistochemistry and other techniques to explore the molecular mechanisms by which the indicators related to the CONUT score (such as lymphocyte subtypes, cholesterol metabolites) affect LNM. Third, compare the combined application value of different nutrition-inflammation scores in the prognosis assessment of breast cancer and construct a more accurate risk prediction model.

## 5 Conclusions

Abnormal nutritional status determined by the CONUT score is associated with LNM in female patients with breast cancer, but NPS not. It suggests that CONUT score may be considered a potential nutritional indicator related to LNM in breast cancer patients. Clinicians can monitor the CONUT score to promptly identify patients with abnormal nutritional status and provide targeted nutritional support treatment, thereby improving the patient’s nutritional and immune status and possibly improving prognosis. Further prospective studies are required to verify its predictive value and clinical application in female breast cancer patients. As for NPS, its relationship with LNM in breast cancer needs to be further clarified by more prospective and multicenter studies.

## Data Availability

Publicly available datasets were analyzed in this study.
